# Knowledge of Nairobi East District Community Health Workers concerning HIV-related orofacial lesions and other common oral lesions

**DOI:** 10.1186/1471-2458-14-1066

**Published:** 2014-10-11

**Authors:** Lucina N Koyio, Wil JM van der Sanden, Elizabeth O Dimba, Jan Mulder, Andre JAM van der Ven, Matthias AW Merkx, Jo E Frencken

**Affiliations:** Department of Primary Health Care Services, Ministry of Public Health and Sanitation, P.O. Box 30016, Nairobi, Kenya; Department of Global Oral Health, College of Oral Sciences, Radboud University Nijmegen Medical Centre, Nijmegen, The Netherlands; Department of General Internal Medicine, Radboud University Nijmegen Medical Centre, Nijmegen, the Netherlands; Department of Oral and Maxillofacial Surgery Nijmegen, Radboud University Nijmegen Medical Centre, Nijmegen, The Netherlands

**Keywords:** Community health workers, Knowledge, HIV-related orofacial lesions, Kenya, Dental caries, Oropharyngeal candidiasis, HIV patients

## Abstract

**Background:**

Human immunodeficiency virus (HIV) related orofacial lesions (HROLs) impact negatively on the health of patients and could be managed at primary healthcare (PHC) level. Community health workers (CHWs) are crucial in optimal patient management through patient identification, education and early referral for professional care. The study objective was to assess knowledge of Nairobi East district CHWs regarding HROLs and other common oral diseases.

**Methods:**

Of the total population of CHWs, 815 [94.5%] completed a 56-item questionnaire covering 5 topics: general dental knowledge, knowledge about HROLs, past encounters with HROLs, current care at community level, opinions regarding oral health problems; and items concerning background characteristics and past training activities. Confirmatory factor analysis revealed Cronbach’s alpha coefficient values of 0.45, 0.59, 0.79, 0.50 and 0.09 respectively. The first four topics were confirmed as domains. Mean minimum score was 0 and mean maximum score was 1 for each variable. However, for ‘past encounters with HROLs, the minimum score was 0 and maximum score was 5.

**Results:**

CHWs had moderate knowledge about general oral health (mean = 0.47) and HROLs (mean = 0.43). None had been formally trained in oral health aspects. Although they had high opinions regarding their role in identifying, educating and referring patients with HROLs (mean = 0.80) to the health facilities, they actually rarely referred such patients.

**Conclusions:**

CHWs need training for building competence in promoting oral health among general and HIV patients in their communities and in early identification and management of non-HIV oral lesions.

**Electronic supplementary material:**

The online version of this article (doi:10.1186/1471-2458-14-1066) contains supplementary material, which is available to authorized users.

## Background

Human Immunodeficiency Virus (HIV) related orofacial lesions (HROLs) are common indicators of HIV infection [1–3]. They are included in the WHO presumptive clinical criteria for HIV infection diagnosis, because of their typical clinical appearance [4]. The lesions may be painful and at times persist for long periods, leading to (prolonged) compromised food intake and exacerbation of the ill-health of patients. These lesions may also cause social discomfort, particularly because HIV infection is accompanied by social stigma.

Being associated with clinical and various immunological stages, the lesions are markers of HIV infection and of HIV disease progression [4], thus facilitating early identification of HIV-infected people and prognosis of those receiving treatment with highly active antiretroviral therapy (HAART) [5–7]. Identifying the lesions at an early stage, to enable optimal patient management, is therefore a necessity.

In Kenya, primary healthcare (PHC) providers (mainly clinical officers and nurses) at health facilities and community health workers (CHWs) in the field are required to integrate oral care of HIV patients into the PHC system [8, 9]. However, these health workers have not been educated on oral diseases. Detecting HROLs as part of their community duties would increase the probability of early identification of HIV-infected people and those developing HAART resistance. In Kenya, HIV patients might not seek early care, even when the signs and symptoms are overwhelming, owing to social stigma and ignorance about the lesions (LNK personal communication).

In line with the national oral health policy objectives of Kenya, of developing targeted community oral health programs [8], a CHW training program on oral care of HIV patients was needed. However, for development of a tailor-made training programme for CHWs and to ensure their participation in the field after completion of the training, it was essential that information first be obtained from CHWs regarding their knowledge and opinions about HROLs and common oral diseases. This study aimed to assess the knowledge and opinions of CHWs regarding HROLs and other oral diseases.

## Methods

Approval for the study was obtained from the Kenyatta National Hospital/University of Nairobi Ethics and Research Committee (approval number KNH-ERC/A/474) and from the Ministry of Public Health and Sanitation (Ref. No. MPHS/IB/1/14 Vol. III). Additional written approval was granted by the Nairobi Provincial Medical officer and the respective district heads for the study to be conducted in Nairobi East district, where a linked study for PHC workers was ongoing [10]. This large rural urban district has a population of nearly 1.4 million [11]. Its multi-ethnic communities have variable literacy and income levels, which are known determinants of CHW performance [12, 13]. The study is registered at the Netherlands Trial Register (NTR2627) and the study protocol was published earlier [14].

### Sampling of the CHWs

The investigation was conducted in the Njiru and Makadara divisions of Nairobi East district from September 2009 through to December 2010. All 430 CHWs from all 4 community units attached to the 4 participating health facilities (HFs) in Njiru division and all 432 CHWs from the 4 CUs in the 4 HFs in Makadara division were invited to participate. In accordance with the newly introduced government guidelines on community health strategy [15], the CHWs had been democratically elected in meetings held by a chief (called *Barazas*), with full participation of the community, community elders, members of the district health management team (DHMT) and community health extension workers (CHEWs). To prevent any confounding or bias, all 862 CHWs were considered eligible for inclusion in the study. This number was adequate for the planned factor analysis procedure [16].

### Development of the questionnaire

A team of three experienced researchers formulated a 56-item questionnaire, after interviewing CHWs and studying the literature [10, 12, 17, 18]. The first section of the questionnaire contained ten items covering background characteristics of the CHWs, like gender, age, level of education, geographical location, length of service as a CHW, name etc. The second section aimed to assess CHW knowledge on oral health topics, starting with the formulation of questions regarding their knowledge of general oral health and of HROLs. In addition, the researchers identified a total of thirteen HROLs, retrieved from four publications [1, 2, 6, 19]. These were: oral candidiasis, hyperpigmentation, enlarged parotid gland, oral Kaposi’s sarcoma, necrotizing ulcerative gingivitis, oral hairy leukoplakia, herpes simplex lesions, oral warts, herpes zoster, molluscum contagiosum, facial skin eruptions, vermilion ulceration and atrophic oral mucosa. Periodontal diseases and tooth cavities, being major oral problems in the general community [20], were also included.

The first author, who is a senior District Medical Officer of Health, spearheaded two focus group discussions and informal conversations with community members, to elicit additional qualitative information from the target community. Each focus group comprised eight community members from HIV support groups (HIV-infected community members who regularly meet at the linking health facilities for psychosocial support and for income generating activities), two community members, two PHC providers trained in recognising HROLs and one district health promotion officer. The focus group members were subjected to viewing 24 slides of HROLs on a computer screen. They identified four lesions that they commonly saw or experienced in the general community. These lesions were oral candidiasis (pseudomebraneous, erythematous and angular cheilitis types), herpes zoster, parotid enlargement and recurrent oral ulcers. They also identified periodontal diseases and tooth cavities as common oral conditions present in their community. These lesions formed the basis upon which questions regarding assessment of CHW knowledge about HROLs and general oral health were constructed.

The focus group discussion also revealed opinions regarding oral health problems such as causes of dental caries and periodontal diseases, and whether CHWs think that it is their responsibility to refer patients with oral problems to the HF. Furthermore, the focus group discussions revealed questions related to past encounters with, and experiences of, HROLs in the community. Finally, focus group members identified aspects that CHWs should know about the treatment of oral lesions and told how they currently manage these lesions.

#### Identification of domains

Five domains of assessment of CHW knowledge regarding HROLs and other common oral lesions were constructed. For each domain, a number of questions were formulated: a) general oral health knowledge (n = 11); b) knowledge of HROLs (n = 19); c) past encounters with experience of HIV-related orofacial lesions in the community (n = 5) d) current care at community level (n = 5) and e) opinions regarding oral health problems (n = 4). The 5 domains consisted of 18 questions, which contained 44 items. Of these, 18 items were closed-ended while 26 were open-ended. In addition, past training in oral health (n = 2) was included as were questions about background characteristics (n = 10). The questionnaire is presented in Additional file 1.

The research team agreed on the correct response to each question/item. In a case of disagreement, consensus was reached after a textbook or recent literature was consulted. For assessment of the open-ended questions, the correct responses were translated into codes after a code structure had been created.

#### Validating the questionnaire

The first draft of the questionnaire was in English and was assessed for face and content validity in a focus group discussion between sixteen PHC trainers involved in various regular CHW training courses in the district. The questionnaire was read out aloud and participants were asked to pay attention to the proper wording and content of the questions and provide suggestions for improvements. This activity led to a second draft of the questionnaire, which was piloted among eight randomly selected CHWs not involved in the study. The outcomes were discussed and refined during a follow-up focus group discussion under leadership of the first author.

The group translated the refined questionnaire into the local language, Kiswahili as both Kiswahili language and English are widely spoken as lingua franca with varying preference according to ethnicity and literacy levels. Difficult words were simplified and native phrases were used to describe unfamiliar terminologies in order to clarify the questionnaire. Some Kiswahili terminologies were incorporated into the English version. As CHWs come from different part of Kenya, the two questionnaires were used simultaneously for data collection. The Kiswahili version, which was similar in structure to the English version, was pretested in another group of eight randomly selected CHWs and refined accordingly. Table 1 provides descriptions and terminologies used by the community to describe oral lesions, and examples of Kiswahili translation that were incorporated into the final draft of the questionnaire. Because two similar kinds of questionnaire were used, it was not possible to apply the common methodology of back translation.

**Table 1:** **Nairobi East district community description of HIV-related orofacial lesions and other mouth problems**

HIV related orofacial lesion	Community description	English translation
Oral candidiasis (Pseudomebraneous)	Vidonda vyeupe vya mdomo	Wounds that are covered with white coating
	‘Oral thrush’	Oral thrush
	Ugonjwa wa mdomo	Disease of the mouth
Oral candidiasis (Erythematous type)	Kuchomeka mdomo	Wounds associated to burns from hot tea.
Angular cheilitis	Vidonda vya ncha za mdomo	Wounds on the corners of the mouth
Herpes zoster	Malengelenge	Lesions with watery fluid under the skin
	Kuchomeka	Lesions associated with burns from hot water or oil
	Mshipi	A disease which is shaped like ‘a belt’
Enlarged parotid	Kufura kando ya masikio	Swelling around the ears
Tooth cavities	Meno yaliotoboka / yalioooza	Holes in the teeth
Periodontal diseases	Kufura ‘gums’	Swollen or bleeding gums
Various skin eruptions	Ugonjwa wa ngozi	Disease of the skin.

#### Administration of the questionnaire

The questionnaire was administered to the CHWs before a regular training course and after written consent had been obtained from each of them. In line with other regular community trainings and activity groups, CHWs were organised into community units (groups of nearly 50) for easy administration. Therefore, a total of 16 training sessions took place, 8 in each division. The questionnaire lay-out and what was expected of them was explained to each CHW. They were allowed to choose between the Kiswahili and English the versions. They were also given a confidential code and were told that it was not an examination but they should respond to the questions to the best of their ability. The first author supervised the sessions and responded to questions, clarifying any misunderstandings. At the end of the session the filled questionnaires were checked, as far as possible, for completeness and legibility of responses.

### Statistical analyses

Raw data was entered into an Excel file by a research assistant and checked by the first author. To minimize bias, two experienced dentists, who understood both languages, independently coded open-ended responses, which were subsequently translated into numeric codes. The two coders compared their results. Non-corresponding codes were discussed until consensus was achieved. A statistician analysed the data, using SAS software (SAS Institute, Cary, NC, USA).

Confirmatory factor analysis was performed on the questions in the 5 domains, using a standard procedure [21]. Data were used to calculate basic statistics (mean, standard deviation). Domain questions were dichotomized according to wrong (0 point) or correct (1 point) answers. Mean values were calculated per domain: i.e., the total domain score was divided by the total number of domain items. This resulted in a mean minimum score of 0 and a mean maximum score of 1 for the variables ‘general oral health knowledge’, ‘knowledge of HROLs’, ‘current care at community level’ and ‘opinions and beliefs regarding oral health problems. The ‘past encounters /experience of HROLs in the community” variable had a minimum score of 0 and a maximum score of 5. Ages were grouped into three categories: 19–25, 26–50, and ≥ 51 years old.

## Results

### Participants

From a total of 862 CHWs, 47 elderly CHWs who could not read or write were subsequently excluded from participating, leaving 815 (94.5%) CHWs in the sample. There were more female (77.3%) respondents. Eleven point eight percent were in the 19-25-year-old group, 79.2% in the 26-50-year-old group and 9.0% in the elderly age group. Those in HIV support groups (denoting that they had publicly declared their HIV-positive status to the community) comprised 35%. The majority (65.1%) were newly recruited CHWs, while 34.9% had done some community work before. None had received formal training in oral health.

### Reliability of questionnaire

Cronbach’s alpha for knowledge about general oral health, knowledge about HROLs, encounter of HROLs in the community and current care at community level were 0.45, 0.59, 0.79 and 0.50, respectively. Confirmatory factor analysis marginally supported the 5-domain structure specified by the authors. The domain ‘opinions and beliefs in oral health problems’ had a very low Cronbach’s alpha coefficient of 0.09 and was not further considered as a domain. The three questions in this domain were used as single items. Table 2 presents Cronbach’s alpha coefficients, mean domain scores, standard deviation, and minimum and maximum possible scores per domain of the resulting 4 domains

**Table 2:** **Cronbach’s alpha coefficients, mean domain scores and standard deviation, and the number of recognized HROLs** ^**1**^

Domain	Number of items	Cronbach’s alpha	Mean ^2^	Std Dev
General oral health knowledge	11	0.45	0.47	0.14
Knowledge of HROLs	15	0.59	0.42	0.16
Encounter of HROLs in the community	5	0.79	2.00	0.61
Current care at community level	5	0.50	0.59	0.21

^**1**^HROLs = HIV-related orofacial lesions.

^**2**^Mean values were calculated per domain, i.e., the total domain score was divided by the total number of domain items.

### Knowledge about general oral health

The mean score for ‘general oral health knowledge’ was 0.47. Although 72% of CHWs were knowledgeable about issues such as that children under 1 year of age should brush their teeth and that tooth cavities could cause dangers to the children, only 8% knew the correct age for initiating tooth brushing for their children. Some even believed that a toothbrush would damage the children’s gums (mean = 0.33). Most (82%) CHWs were aware that sugary snacks may cause mouth diseases in children but fewer (53%) related it to the development of tooth cavities. Their belief that carious teeth in children should not be extracted (mean = 0.66) was noted.

CHW knowledge concerning the handling of mouth injuries, such as avulsed teeth, was very low. Although 83% of them would refer dental emergencies to the health facility, only 18% would preserve avulsed teeth and refer an injured child to the HF. Similarly, a negligible proportion (0.5%) knew that conditions, such as diabetes and old age, could predispose patients to oropharyngeal candidiasis (OPC). Only 39% of CHWs were aware that diabetic patients could develop OPC.

### Knowledge about HIV-related orofacial lesions

The mean score for ‘knowledge about HROLs’ was 0.59. CHWs were highly knowledgeable about signs and symptoms of OPC (mean = 0.84) and about the fact that HIV infection leads to frequent ulcers in the mouth (mean = 0.74. However, their knowledge of OPC as a clinical sign of HIV infection was lower (mean = 0.47) and was very poor with regard to identifying OPC as a marker of HAART failure (mean = 0.12). It was also noted that CHWs were highly knowledgeable about the fact that patients with OPC should be referred to the health facility (mean = 0.93). Their main reason given about why CHWs referred patients was ‘treatment’. Rarely did they inform their patients about the possibility of HIV testing to ascertain their HIV status.

Of the CHWs, 70% knew at least one sign and symptom of HIV infection in the orofacial region, and the one most commonly mentioned was OPC. Although 67% of CHWs indicated that they knew that herpes zoster could occur in the facial region, only 34% of them could correctly diagnose it in the facial region when the signs and symptoms were described in the questionnaire.

### Past encounter with HIV-related lesions in the community

The mean score for this domain, which included the 3 common OPC clinical presentations (pseudomebraneous, erythematous and angular cheilitis)**,** bilateral parotidomegaly and herpes zoster, was 2.

### Current care at community level

The mean score for this domain was 0.59. Two questions, related to past referral of patients with any mouth lesions to the HF in the previous 3 months (mean = 0.33) and to the type of lesions that were referred (mean = 0.64) had lower scores than the remaining 3 questions that were related to informing patients with mouth problems, including oral candidiasis, that they should visit health facilities (means = 0.70, 0.85 and 0.93).

### Opinions and beliefs in oral health problems

Nearly 63% of the CHWs patients felt they should be able to identify oral thrush in their community. About 13% believed that oral thrush can be caused by witchcraft while nearly all (91%) felt a patient with dry mouth should be referred to the health facility.

## Discussion

Being democratically elected members of the community, CHWs in the present study most probably represented the perceptions of the community regarding oral health in HIV infection. Female dominance could be attributed to the gender roles in this community: female members, as home keepers, are expected to serve within home settings while the male counterparts work outside the community, earning incomes to support their families. This situation was similar to that in a WHO review [22]. That about one third of the CHWs were in HIV support groups meant that they had declared their HIV-positive status to their communities. They, therefore, freely shared their (experiential) knowledge on HIV topics. The option of using either the English or the local language version of the questionnaire, which fellow community members had translated, enabled the CHWs to express their thoughts accurately. This uncommon approach was necessary in this community but prohibited the commonly-used method of back translation.

The questionnaire structure, consisting of various domains, allowed more detailed assessment and interpretation of the knowledge of the CHWs and their willingness to perform the expected tasks. This information was essential in the execution of the planned training. However the Cronbach’s alpha coefficients for all but one domain were lower than the accepted benchmark of 0.7. It is possible that CHWs did not consistently interpret all the questions in the domains, or have a different level of knowledge on specific items. This could also partly due to the low deviation in scores per item (all respondents agree or disagree). Further education on all topics might therefore be necessary.

### General oral health knowledge

Tooth decay is a common oral problem that is preventable through implementing simple oral hygiene measures at home, such as tooth brushing and avoidance of excess sugar intake. Although a moderate portion of CHWs were aware that it was important for children to brush their teeth and that tooth cavities could pose dangers to the children, most did not know the correct age at which to initiate tooth brushing among children. The highest score was found for the item ‘excessive sugar intake is risky to oral health’ which can be attributed to the media marketing campaigns of toothpaste manufacturing companies that alluded to this danger. CHWs, however, had knowledge gaps with regard to the actual dangers of sugar as a risk factor for developing tooth cavities. This was also noted in their incorrect responses to the items ‘sugary foods could cause tonsillitis and mouth ulcers’ and in the incorrect advice that they said they gave to community members with mouth ulcers.

According to the CHWs, mouth injuries and avulsed teeth are common among children in this study population. The successful management of avulsed teeth at the HF would largely depend on the first aid action taken by the community to preserve avulsed teeth and on timely referral of the children to the HF. This study showed that only 18% of CHWs would preserve an avulsed tooth and refer an injured child to the HF. The majority would throw away the tooth and would control the bleeding. It was beyond the scope of the questionnaire to assess how the 18% would preserve the tooth and whether they would treat the injury as an emergency.

### Knowledge about HIV-related oral lesions

The CHW knowledge about HROLs was low, which was probably due to the absence of formal training on these diseases. The result of the present study showed that CHWs had seen common HROLs, such as angular cheilitis, OPC, cheilitis, bilateral parotidomegaly and herpes zoster, which confirmed that the lesions are common in this community. Referral of these patients to the HFs requires that CHWs know about the clinical importance of these lesions and the care available at the HF. Although they had high opinions about their role and about the importance of referring patients with oral lesions to the HFs, the ‘current care’ domain showed that they had rarely referred patients with oral lesions to the health facilities in the preceding months. This probably indicated that the CHWs lacked the competence and skills needed for educating the community and mobilizing it to seek oral healthcare services.

CHWs were most familiar with OPC, probably because it is the commonest lesion in HIV infection. Another reason could be that it is commonly described in regular HIV training courses in Kenya and on educational posters as being an opportunistic infection. CHW knowledge regarding its clinical significance was, however, low. CHWs associated this lesion with allergic reactions to drugs, a dirty mouth, excessive intake of milk, acidic, hot or sugary foods and ‘diseases’ in the stomach. Similar incorrect explanations (including defaulting on immunization) were given with regard to bilateral parotidomegaly. Consequently, the incorrect advice was given to community members with OPC; such as avoidance of nutritious foods like milk and fruits. A common practice of concern, mentioned by the CHWs, was that traditional healers in the community provided herbs that could induce vomiting and diarrhea, to ‘clean the stomach’. These factors could explain why only 34% of the CHWs had recently referred oral lesions of community members to the HF, despite their strong opinions on this subject. On the other hand, they also lacked knowledge of non-HIV-related causes of HIV infection. It was further noted that, although 35% of CHWs were in HIV support groups, with expected experiential knowledge, their knowledge of the clinical significance of OPC as a marker of HAART failure was poor. A similar observation of lack of knowledge about OPC was made in an earlier study covering first-line healthcare providers [18].

CHWs failed to diagnose herpes zoster in the facial region despite its characteristic dermatome distribution. The local word ‘mshipi’, meaning ‘a belt’, could indicate that they associated it with the abdominal region. They related signs and symptoms in the face to a skin disease, chicken pox, allergies and burns. This was consistent with another local word, ‘kuchomeka’ meaning ‘burns’, which they used in describing the facial lesions as kitchen burns. Only a handful recognized a conspicuous herpes zoster scar in the facial region of a community member.

### CHW willingness to participate in oral care

Positive opinions of CHWs on identification and referral of patients with oral lesions to the HFs suggest that they were willing to perform oral health promotional tasks in the community. This finding was similar to that of PHC providers in the linking HFs in an earlier study [18]. The willingness to perform oral healthcare activities at both levels of care suggests that introduction of a successful oral health program is a possibility in this setting. Competences of CHWs could be enhanced through: training that would empower them with the knowledge and skills needed for identifying the lesions. Equipping them with oral health promotional materials such as posters and information about available care at the HFs and ensuring that they acquire good communication skills for relaying the messages would also need to be incorporated into their training [12]. Assessing and describing knowledge alone has limited value for designing an intervention, particularly if this is related to past experience with the disease. However, it appears that this is the first study which has investigated current performance of CHWs. Further studies might build on the results and experiences described.

### Study limitations

This study was conducted in a low socio-economic population, which is also not used to answering questionnaires. For example, a number of CHWs could neither read nor write, and were excluded from the study. Moreover, some CHWs may not have understood all the questions, and might have asked for clarification. This might have affected the results, as during clarification of misunderstandings additional information might have been provided, which might have resulted in overestimation of current knowledge. Potential confounding factors were not taken into consideration, as the current study only sought to assess the knowledge of the CHWs, which turned out to be low to moderate. This necessitated the development of a training course for increasing their knowledge in order to attain the aim of the main study.

The generally low Cronbach’s alpha values for the domains indicate low reliability of the questionnaire and the need to further validate this questionnaire. In addition, it should be noted that that two of the domains with Cronbach’s alpha <0.60 had greater than 10 items each, while the domain with the Cronbach’s alpha > 0.70 had 5 items. This is remarkable, and CHWs might not consistently have interpreted all the questions clustered as measures of a particular domain as such. This means, that the measures that produced Cronbach’s alpha coefficients of lower than the acceptable benchmark of 0.70 might be less reliable measures of the underlying construct measured in this study. Just a plain presentation of all single results might also have answered part of our research questions, and development of the purported constructs, which might be less reliable in this situation, would subsequently be redundant.

## Conclusion

This study showed that CHWs were willing to implement oral health promotional activities in their communities but lacked the appropriate knowledge required. In this high HIV prevalence community, CHWs need to be educated about general oral and HIV-related oral diseases, early identification of (HIV-related) oral lesions and referral of community members suspected of being HIV-positive to the HFs.

## Electronic supplementary material

Additional file 1: **Questionnaire.** (PDF 204 KB)
