# Supplementary material for: Knowledge of Nairobi East District Community Health Workers concerning HIV-related orofacial lesions and other common oral lesions
Source: BMC Public Health. 2014 Oct 11;14:1066. doi: 10.1186/1471-2458-14-1066 (PMC4201672; doi:10.1186/1471-2458-14-1066)
Supplement: Supplementary file 1 — Additional file 1: Questionnaire. (PDF 204 KB) [file 12889_2014_7161_MOESM1_ESM.pdf]

AIM OF THE QUESTIONNAIRRE:

To assess CHWs' opinions, knowledge and actual care on management of oral lesions:

- Knowledge of oral health care at community level
- Knowledge on prevention of common oral diseases such as dental caries and periodontitis at the community level.
- Knowledge of common oral problems of HIV patients.
- The importance of recognition of suspected oral HIV lesions at the community level and referral to the health facility.
- Recognition of the clinical appearance of various oral HIV lesions

  Pseudo membranous Candidiasis, Erythematous candidiasis, Angular Cheilitis, Parotid enlargement, Herpes Zoster
- The knowledge of oral health services that can be provided at the health facility

THIS IS NOT AN EXAMINATION

Please fill in the following details

**NAME**<br>(jina)	
**The identification number you were given**	
**Sex (please circle one)**	**MALE / FEMALE**
**Age**	**_____________ years**
**Level of education(circle one)**	**Primary school**<br><br>**Secondary school**<br><br>**College**
**Community Unit**<br>(Eneo lako la huduma ya afya)	
**When enrolled as a Community Health Worker**<br>(Wakati ulianza kuwa mhudumu)	________________ ________________<br>**Month** (Mwezi) **Year** (Mwaka)
**Marital status(circle one)**<br>(Hali ya kuoa/kuolewa)	**Single**<br><br>**Married**<br><br>**Widow/ Widower**<br><br>**Divorced**
**Are you a member of a HIV support group? (circle one)**	**Yes** **No**

THIS IS NOT AN EXAMINATION. PLEASE DO NOT GUESS. IN CASE YOU DO NOT KNOW THE ANSWER TO A QUESTION WRITE "I DON'T KNOW"

THIS IS NOT AN EXAMINATION

**1.**	**Please circle either,** I *strongly agree* or I *agree* or *I don't agree* or *I strongly disagree* **to each of the following responses.** **Example**				
		I agree	I don't agree	I strongly disagree	Nairobi is the capital city of Kenya
	I strongly agree	I agree	I don't agree	I strongly disagree	A community health worker should be able to identify oral thrush (*ugonjwa wa mdomo*) among his/her community members
b)	I strongly agree	I agree	I don't agree	I strongly disagree	Children with tooth cavities should have their teeth removed because they will soon grow permanent teeth.
c)	I strongly agree	I agree	I don't agree	I strongly disagree	Oral thrush(*ugonjwa wa mdomo*) causes red painful wounds in the tongue and throat
d)	I strongly agree	I agree	I don't agree	I strongly disagree	Children under 1 year should not brush their teeth because the toothbrush will damage their gums
e)	I strongly agree	I agree	I don't agree	I strongly disagree	Most people that smoke develop white patches in the mouth.
f)	I strongly agree	I agree	I don't agree	I strongly disagree	People with diabetes develop oral thrush (*ugonjwa wa mdomo*).
g)	I strongly agree	I agree	I don't agree	I strongly disagree	Herpes zoster(*mshipi au malengelenge*) also occurs in the face and mouth region
h)	I strongly agree	I agree	I don't agree	I strongly disagree	HIV infection causes frequent wounds in the mouth (HIV infection husababisha vidonda vya kila mara mdomoni)
i)	I strongly agree	I agree	I don't agree	I strongly disagree	Oral thrush (*ugonjwa wa mdomo*) is in most cases a sign of HIV infection

2. **a) At what age should mothers in your community start to brush the teeth of their children?**

3. **Mouth problems** (ugonjwa wa mdomo) **are common among HIV patients.**

a. Please write down maximum three signs and symptoms you know of HIV infection in the mouth and face region (taja njia tatu ambazo ugonjwa wa mdomo hujitokeza kwa uso au mdomo wa mgonjwa mwenye virusi)

i)

ii)

iii)

	b.	Oral thrush (*ugonjwa wa mdomo*) can be caused by witchcraft (please circle one)<br>I strongly Agree I agree I don't Agree I strongly Disagree
	e.	What advice do you usually give to your community member who develops oral thrush?
	f.	In your opinion, please write down a maximum two kinds of services the doctor at your health facility will give to a patient with oral thrush?
		i)
		ii)
**4.**	**Lillian, a 12 year old child in your community unit has just fallen down while playing. You notice one tooth has come out** (jino limeng'oka lote)	
	a.	According to your opinion, what should the mother do with the tooth?
	b.	According to your opinion, what should the mother do with the child?
**5.**	**Kamau, the shopkeeper, in your community unit mainly sells juices and biscuits to the school children over break time.**	
	a.	Do you think these school children are exposed to mouth diseases? (Circle one)<br>Yes, very much Yes, a little bit No I don't know
	b.	If your answer to the above question is yes, which mouth disease do you think, might the children be exposed to?
**6.**	**Janet, a 23 year old mother on ARVs from your community unit, has not been eating well because of very painful wounds in her tongue.**	
	a.	Why, according to your opinion, do some patients on ARVs develop wounds in the mouth?
	b.	What will you advice Janet to do?

THIS IS NOT AN EXAMINATION

	**HAVE YOU EVER SEEN THE FOLLOWING MOUTH PROBLEMS IN YOUR COMMUNITY? WHAT DO YOU KNOW ABOUT THEM?**		
**7.**	**The patient has wounds at the corners of the mouth making it painful to open the mouth**		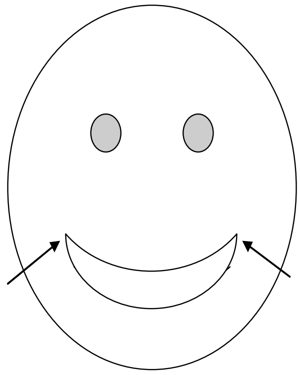
	a.	I have seen such a mouth problem in my community<br>Yes, very often / Yes, But not often / No / I am not sure	
	b.	I think, this mouth problem is caused by:<br>i)	
		ii)	
**8.**	**The patient develops skin rashes and blisters which have watery liquid under the skin** (ngozi inakuwa na maji ndani) **on one side of the face. The water breaks to form painful wounds which take long to heal** (maji hupasuka na kuacha vidonda. Vidonda huchukua muda mrefu kupona).		
	a.	I have seen such a mouth problem in my community<br>Yes, very often / Yes, but not often / No / I am not sure	
	b.	I think, this problem is called	
**9.**	**The patient develops wounds on the tongue and throat. The wounds are covered with a white substance**(vidonda vyenye rangi nyeupe). **Eating and swallowing usually is painful. Both adults and children can be affected.**		
	a.	I have seen such a mouth problem in my community<br>Yes, very often / Yes, but not often / No / I am not sure	
	b.	I think, this mouth problem is caused by:	

THIS IS NOT AN EXAMINATION

**10.**	**The patient develops a big soft swelling on both sides around the** ears (kufura hasa karibu na masikio). **Common in children but is also seen in adults.**	
	a.	I have seen the problem in my community<br><br>Yes, very often — Yes, but not often — No — I am not sure
	b.	I think, such a problem is caused by:
		i)
		ii)
**11.**	**The patient develops red wounds in the mouth which may make eating and swallowing painful. They can be on the tongue or throat. They affect both adults and children.**	
	a.	I have seen such a mouth problem in my community<br>Yes, very often — Yes, but not often — No — I am not sure
	b.	I think, this mouth problem is caused by
**12.**	**Give maximum two reasons why, according to your opinion, all patients with oral thrush**(ugonjwa wa mdomo) **should be referred to the health facility immediately**	
	a.	
	b.	
**13.**	**Please write down maximum 3 mouth diseases that, according to your knowledge, are commonly associated with HIV infection.**	
	a.	
	b.	
	c.	

THIS IS NOT AN EXAMINATION

**14.**	**Wahome, a 68 year old elderly diabetic *mzee* in your community unit developes painful wounds in the mouth which are covered with a white substance. He asks you as a community health worker what the wound could be.**	
	a.	Why do you think he developed the wounds?
	b.	What advice would you give Wahome?
**15.**	**HIV patients sometimes develop dry mouth** (mdomo kukauka) **as a result of decreased flow of saliva** (kupunguka mate).	
	a.	What advice do you normally give to a patient with dry mouth at community level to increase saliva flow?
	b.	Please write down maximum 2 dangers of dry mouth
		i)
		ii)
	c.	Do you think that a patient with dry mouth should be referred to the health facility?<br>Yes, I strongly think so Yes No I do not know
**16.**	**During your health talk to the mothers in the community unit, the mothers ask you why tooth cavities might be dangerous.**<br><br>**Please fill in the spaces below, maximum 3 reasons why tooth cavities might be dangerous, according to your knowledge.**	
	a.	
	b.	
	c.	

THIS IS NOT AN EXAMINATION

**17.**	**How many patients with HIV suspected mouth diseases have you referred from your community unit to the health facility in the last three months?**	
	a.	No, I have not referred any patients with mouth problems from the community to the health facility.
	b.	I have referred about___________________ patients with mouth problems from the community (Please fill this space) to the health facility
**18.**	**Which type of mouth diseases did you commonly refer from the community to the health facility in the last three months?**	
		i)
		ii)
		iii)
		iv)
**19.**	**Have you ever received training on mouth health care of patients at community level?**	
	a.	Yes No (please circle your answer)
	b.	If yes, please explain

THANK YOU FOR

ANSWERING THESE QUESTIONS
